# Supplementary material for: Generating giant and tunable nonlinearity in a macroscopic mechanical resonator from a single chemical bond
Source: Nat Commun. 2016 May 26;7:11517. doi: 10.1038/ncomms11517 (PMC4894958; doi:10.1038/ncomms11517)
Supplement: Supplementary Information — Supplementary Figures 1-10, Supplementary Tables 1-2, Supplementary Notes 1-3 and Supplementary References [file ncomms11517-s1.pdf]

Supplementary Figure

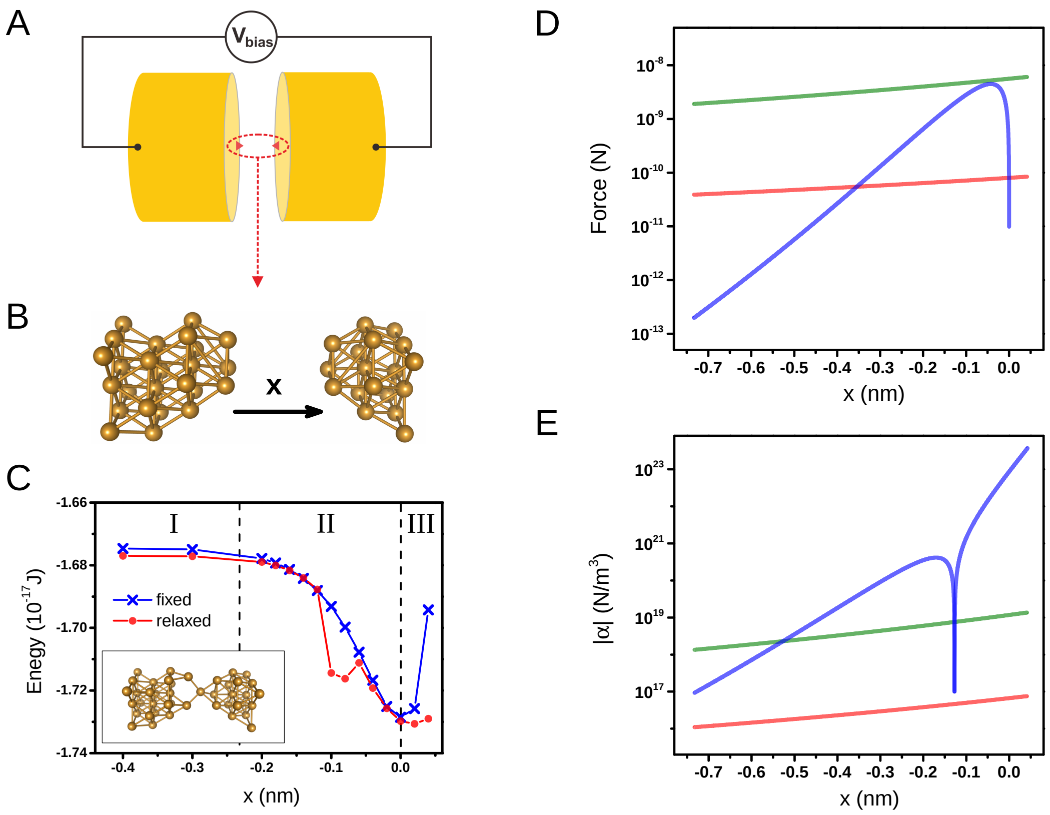

Supplementary Figure 1 | Theoretical modelling of the nonlinearity. (**A**)The structure of the model of atom contact includes a pair of macro-scale cylinders with transversal area of diameter of d, and a pair of atom clusters (red triangles) in the middle of the surface.**B**) Sketch of the clusters used in the DFT simulation. The relative atom position in each cluster is fixed while varying the displacement x of right cluster with respect to the left one. (**C**) The variation of the system's total energy with the displacement x for the cases of fixed and relaxed atom positions. For the case of the relaxed one, gold atom relocation can happen, which leads to a jump in the system's energy. Inset indicates such a process, where a single gold atom jumps to connect the two clusters. (**D**), Typical attraction force between the contact, green for vdW, red for electric static force (the bias being 200 mV), and blue for short range chemical bonding force from DFT calculations, with diameter d being set to 30 nm, similar to experimental condition. (**E**) same as **D** but for the strength of nonlinear response α.

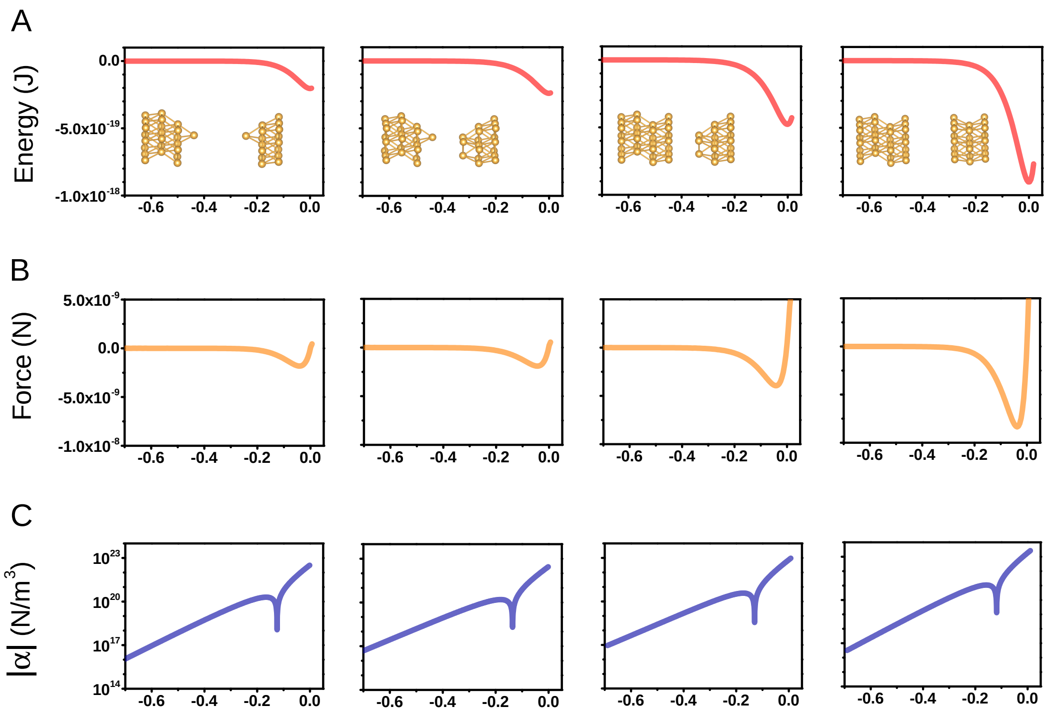

Supplementary Figure 2 | Simulation results for different atom structures. (**A**) DFT results of interaction energy of different atom structure as a function of x, with the clusters shown in inset. (**B**) same as (**A**) but for the force. (**C**), same as (**A**) but for the amplitude of Duffing nonlinear response.

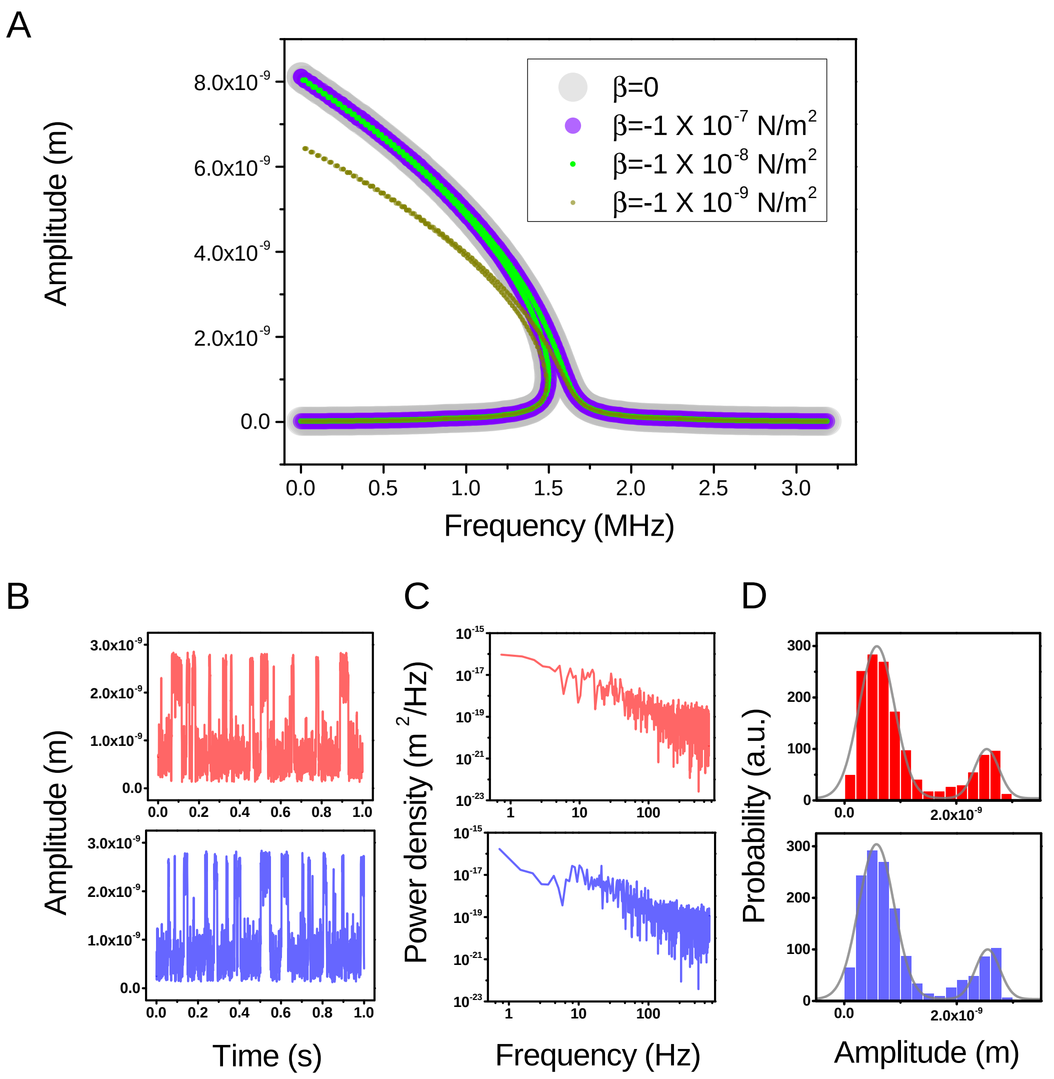

Supplementary Figure 3 | Simulated effects of quadratic nonlinearity. (**A**), the frequency response under coherent drive with quadratic nonlinear response $\beta = 0$ (light grey), $-1 \times 10^7 \mathrm{N/m^2}$ (purple), $-1 \times 10^8 \mathrm{N/m^2}$ (green) and $-1 \times 10^9 \mathrm{N/m^2}$ (olive). The Duffing nonlinearity coefficient is $\alpha = -2 \times 10^{17} \mathrm{M/m^3}$, and other parameters are close to those for our device. (**B**) , Fluctuation induced bi-states dynamics with $\beta = -1 \times 10^7 \mathrm{N/m^2}$ (red) and $\alpha = -2 \times 10^{17} \mathrm{M/m^3}$, and other parameters are close to conditions of the device in Fig 4 in main text. Blue dot, same as red but for a different coefficient $\beta = 0$. (**C** and **D**), power spectrum density and amplitude distribution of the bi-states corresponding to (**B**).

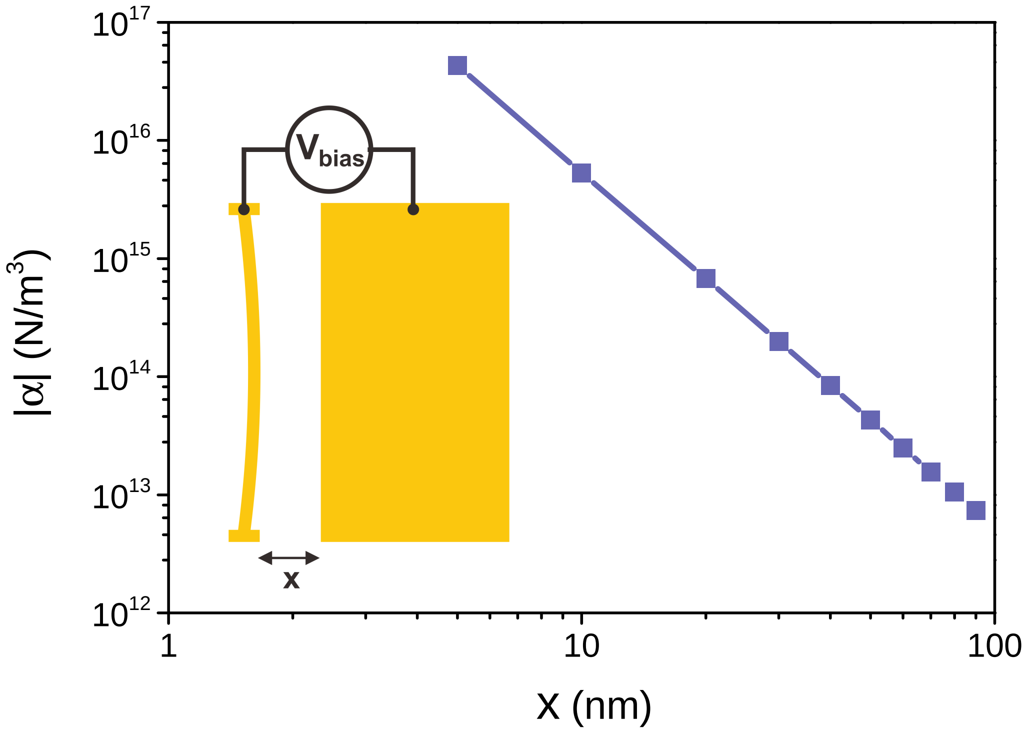

Supplementary Figure 4 | Estimation of the nonlinearity induced by the electrostatic force. The doubly clamped beam same as the one used in our experiment is employed, the voltage bias is adaptively chosen so that maximum nonlinear response is reached while the beam is not attracted to touch the electrode, and the minimum gap is set at 10 nm for the consideration of practical fabrication.

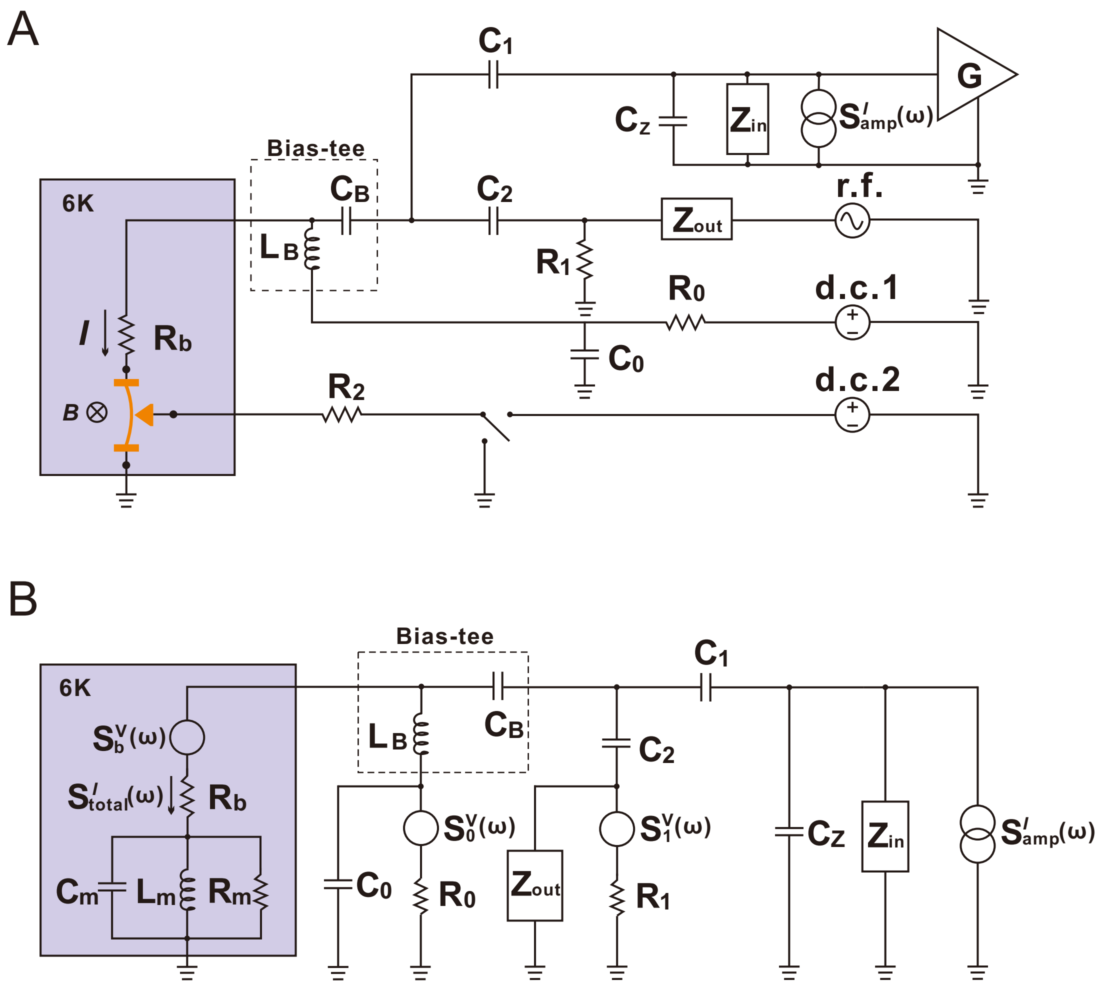

Supplementary Figure 5 | Measurement circuit for experimemt. (**A**) Scheme of excitation and detection. (**B**) Equivalent circuit used to estimated electronic noise of the experiments. All the elements, such as resistances, are Thévenin equivalent to the white noise source of voltage. Room temperature is 300 K and the device temperature is 6 K. Inputs of the preamplifier are equivalent to a voltage noise and current noise source to describe the imprecision and the back-action, with the corresponding strengths being experimentally measured. The r.f. signal is modeled as an ideal driving plus a white voltage source whose strength is calculated from the phase noise.

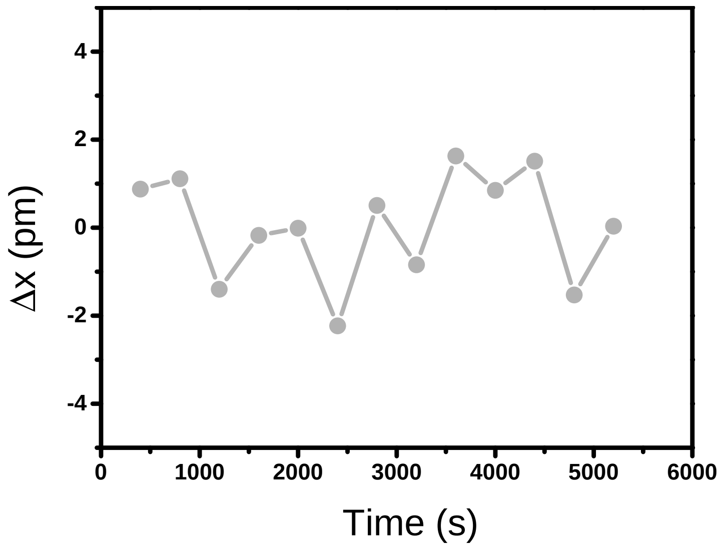

Supplementary Figure 6 | Displacement stability of the device. Measured by recording resonance frequency drift of the beam for a typical device. To do this, we pull the beam to the position where a significant force-frequency dependence is observed. The amplitude is kept below $50\ \mathrm{pm}$, so that the system is in linear regime. the measured standard deviation is $1.2\ \mathrm{pm}$.

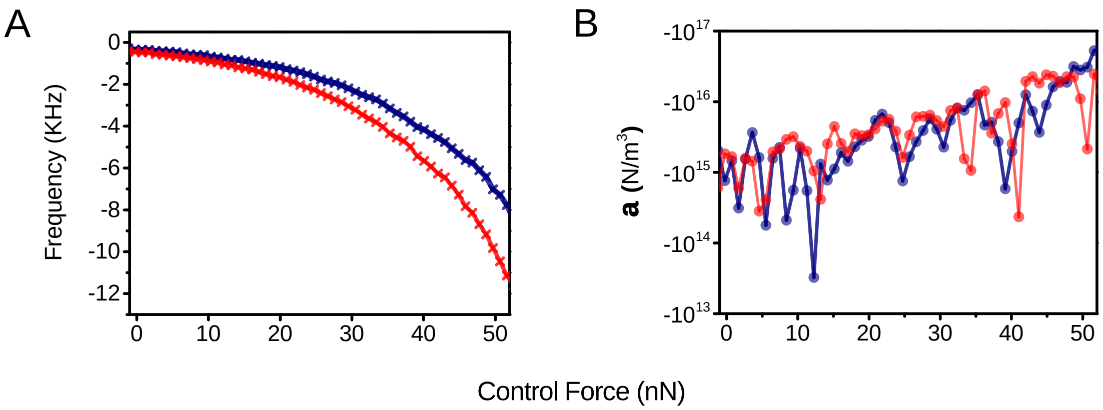

Supplementary Figure 7 | Reversibility of the device. (A). The frequency shift as a function of control force F in non-contact regime before (Red) and after (Blue) jump-to-contact for a typical device. **(B)**. Same as **(A)** but for Duffing nonlinear response α.

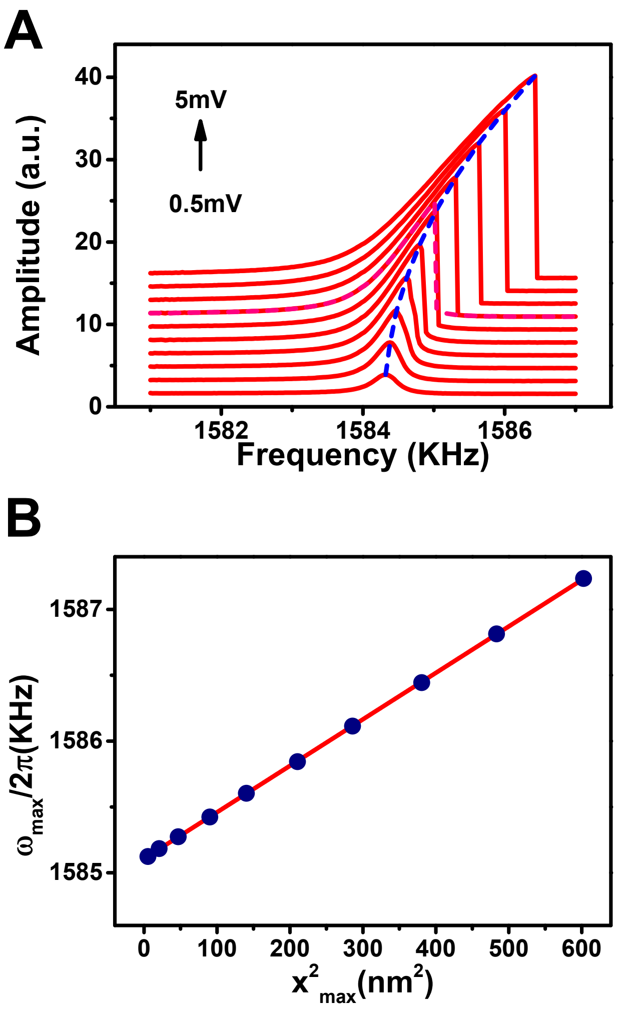

Supplementary Figure 8 | Intrinsic nonlinearity of the beam. (**A**) Frequency response of the beam under different driven strength for a typical device. The frequency is swept from low frequency to high frequency. The dashed line indicates the position of the peak. The Data are measured by pulling the beam far away (larger than 30 nm) from the stiff electrode. (**B**) Peak frequency as a function of the corresponding square of vibration amplitude. The data are linearly fitted to obtain the nonlinearity.

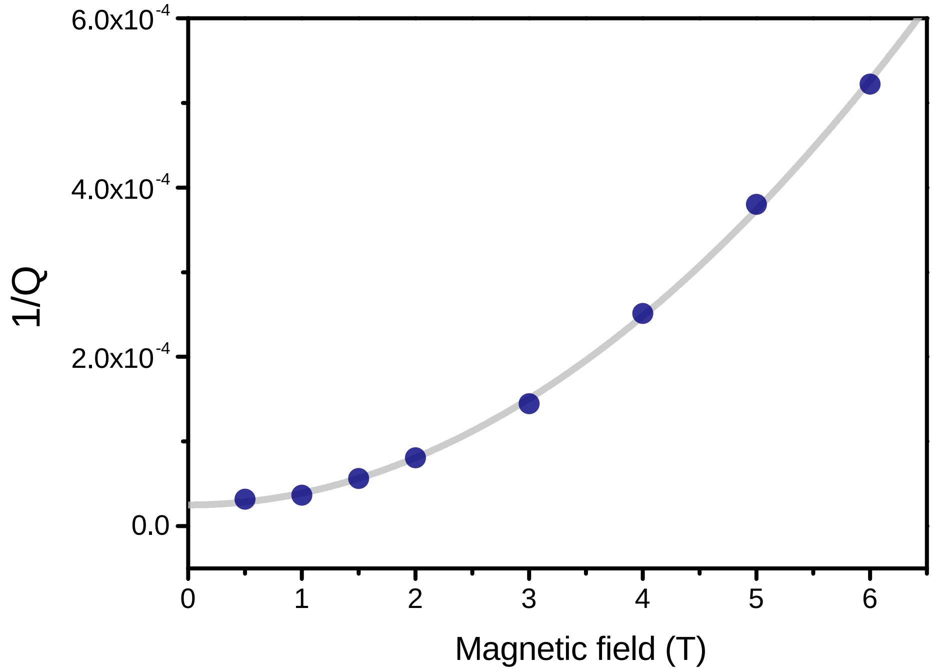

Supplementary Figure 9 | Magnetic field induced damping Measured quality factor Q as a function of magnetic field for a typical device, with gray line being quadratic fit. The intrinsic quality factor obtained from fits is about 40,000.

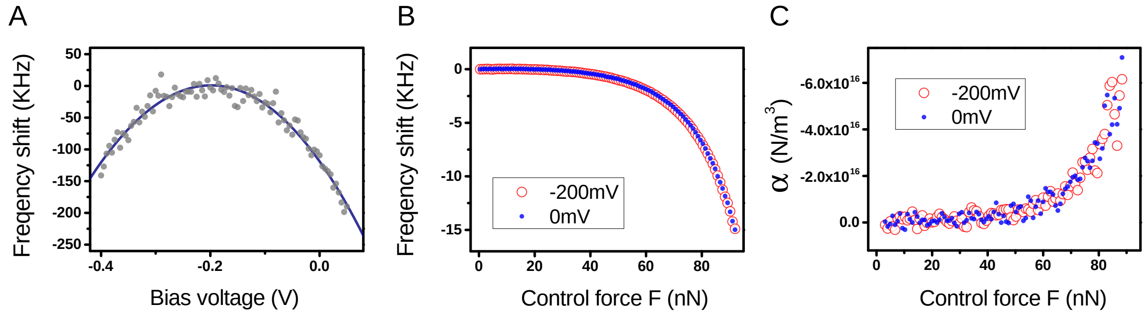

Supplementary Figure 10 | Electrostatic force effects. (**A**) Electrostatic force induced frequency shift is measured in non-contact regime for a typical device by fixing the control force and changing the bias voltage on the stiff electrode. The data are fitted to quadratic function and are peaked at -200 ± 10 mV. (**B, C**) The frequency shift (**B**) and Duffing constant α (**C**) as functions of the applied external force for two bias voltages of 0 mV and -200 mV.

Supplementary Table

Supplementary Table 1 | Comparison of reported mechanical nonlinearity

System	$\alpha(\mathrm{N/m^3})$	Generation mechanism	Tuning methods
Our Method	1.1×10^{20}	Short-range chemical force	Mechanical
Qubit-coupled resonator [1]	8×10^{15}	Qubit charge interaction	Electrical
Mechanical-Electrostatic resonator [2]	5×10^{14}	Electrostatic force	Electrical
Mechanical-van der Waals resonator [3]	2.0×10^{9}	Van der Waals force	Mechanical
Mechanical-Casimir resonator [4]	3.0×10^{9}	Casimir force	Mechanical
Opto-mechanics [5]	6.6×10^{8} *	Optical force	Optical
Traditional micro/nano-resonator [6]	7.6×10^{15} **	Intrinsic tension	–
Bottom-up resonator [7]	1.9×10^{16} **	Intrinsic tension	–

*: intracavity photon number $n_\gamma \approx 1.0 \times 10^7$, **: represented high value

Supplementary Table 2 | Noise Comparison

Noise Source	Strength
Mechanical Brownian Noise	$330\ \mathrm{aN \cdot Hz^{-1/2}}$
Back-action Current of amplifier	$51\ \mathrm{aN \cdot Hz^{-1/2}}$
Phase Noise of r.f. source	$46\ \mathrm{aN \cdot Hz^{-1/2}}$
Electronic Johnson-Nyquist noise	$30\ \mathrm{aN \cdot Hz^{-1/2}}$
Electro static force	$< 20\ \mathrm{aN \cdot Hz^{-1/2}}$
Tunneling current noise	$< 1\ \mathrm{aN \cdot Hz^{-1/2}}$

Supplementary Note 1 | Theoretical calculations of the system

Calculation of long range interaction

The atom contact interaction is modeled by considering both long-range and short-range chemical interactions with the geometry shown in Supplementary Figure 1. The long-range force is estimated by considering the van der Waals (vdW) attraction and electrostatic force, and the geometric model is two gold cylinders close to each other, similar to the case of our nano-bridge structure, as shown in the Supplementary Figure 1A .

The vdW has the expression of the form [8]:

$$F_{\text{vdW}}(x) = -\frac{HS}{6\pi x^3}, \tag{1}$$

where H is the Hamaker constant taking the value 4×10^{-19} J for gold, S is the cylinder transversal area and x is the separation of the atom contact.

The Electrostatic force is calculated as:

$$F_{\text{elec}}(x) = \frac{\varepsilon_0 V_{\text{bias}}^2 S}{2x^2}. \tag{2}$$

with V_{bias} the effective voltage bias on the gold contact. The total long-range forces $F_{\text{long}}(x) = F_{\text{vdW}}(x) + F_{\text{elec}}(x)$ can lead to change of the mechanical property of the resonator.

Density functional theory calculations of the short range chemical bonding force

To simulate the chemical bonding force of the gold-atom contact, we have performed first-principle calculations based on Density functional theory (DFT) method by using the Vienna ab initio simulation package (VASP)[9]. An atom contact is supposed to be fixed at the surface of a nanobridge as sketched in Supplementary Figure 1B, the atom contact is simplified to be two sub-nanometer-sized gold-atom clusters that are separated by a distance x. A thick-enough vacuum space along the x direction is used to eliminate the interactions between the atom contact and those in image unit cells. This model captures the mechanical feature of the experimental setup which is dominated by the interaction between the first-layer atoms near the contacting regime.

The generalized gradient approximation (GGA) proposed by Perdew, Burke and Ernzerhof [10] is adopted for the exchange-correlation functional. The projected augmented wave method[11, 12] with a plane-wave basis set is used. To improve the precision of the calculated total energy, a relatively large plane-wave cutoff energy 1.5×10^{-16} J is used. The convergence criterion of the system total energy is 1.6×10^{-24} J. Because of the large size of the unit cell and the vacuum region, Γ-point sampling is adopted for the Brillouin zone.

Firstly, the system of the two clusters with a relative separation of $x = 0.8\ \mathrm{nm}$ in between is fully relaxed until the total force reaches the criterion of $3.2\times10^{-20}\ \mathrm{J}\cdot\mathrm{nm}^{-1}$. Then the system's total energy for different x is calculated by varying the distance x with all the gold atoms in each cluster fixed. The obtained total energy of the system is shown in Supplementary Figure 1C. When the distance between the two clusters decreases, the variation of the system's total energy can be divided into three regions, as marked as region I, II, and III in Supplementary Figure 1C. In region I, where the distance is very large, the total energy remains almost constant as a result of weak interaction between the two clusters. In region II, total energy becomes lower, indicating that the attractive interaction emerges as the distance decreases. In region III, the total energy quickly increases with decreasing distance, showing that when the distance being sufficiently small, the interaction between the two clusters changes from attraction to repulsion.

In principle, the relative positions of the contacting gold atoms in each cluster may change when x changes. To simulate this, we also repeat the calculation by allowing the positions of the contacting gold layers to relax. As shown in Supplementary Figure 1C, in region I the total energy of the relaxed system remains nearly constant, with a slight energy shift from that of the fixed system. In region II, the total energy of the relaxed system varied dramatically in a certain range. As shown in the inset of Supplementary Figure 1C, in this region one of the surface gold atom of the left cluster is pulled into the middle, which significantly lowers the system total energy and alters its mechanical property. In practice, the vibration amplitude is very small, so significant changes in atom positions would not happen. For qualitative analysis, we adopted the total energy of the fixed system in simulating its mechanical property.

Due to system errors in performing DFT calculations, the 2nd order derivative of the total energy shows a slight oscillatory behavior at large distance range. We smooth the calculated data by fitting it to $y = \frac{A}{x^a} + \frac{B}{x^b}$. In this way, the nonlinearity coefficient, which relates to the 4th order derivative of the total energy, is qualitatively evaluated based on the fitted data.

We compare the long-range and short-range forces (Supplementary Figure 1D) and the corresponding Duffing nonlinear response α (Supplementary Figure 1E) as a function of x, the separation of the atom cluster. The corresponding separation of the cylinders is taken the thickness into x_{thick} account as $x + x_{\mathrm{thick}}$, $x_{\mathrm{thick}} \approx 0.84$ nm equals to thickness 7 layers of atom used in our model. Though the strength of force of long-range interaction can be comparable to that of short-range interaction, however, the nonlinear due to the former is much weaker than the latter, as expected.

In practical application, atom distribution in the cluster can not be precisely controlled, so we also perform calculations by using various different atoms distributions, the results are shown in Supplementary Figure 2. The resulted strengths of nonlinear response α is found to have similar character with high nonlinear response.

Model of the jump-to-contact

The chemical force can be used in two regimes. One is the contact regime where metallic bonds between atom contact are full formed. In this regime, the system is stable unless significant high

force are employed to break the bonds. The other working regime is attraction regime where electron wave functions from the two clusters of the atom contact are partially overlapped. In this regime, the system is relatively unstable and the external excitation can induce the system to jump to contact. To estimate such a process, we notice that the force of chemical bond has the characteristic that when breaking (or formation) a bond, there is a point where the attraction has the maximum value. This point corresponds to the zero point of the second order derivative of the chemical energy $U_{\text{chem}}(x)$, which is denoted as x_n. We have

$$-\frac{\partial^2 U_{\text{chem}}}{\partial x^2}\bigg|_{x=x_n} = 0 \tag{3}$$

and

$$-\frac{\partial^3 U_{\text{chem}}}{\partial x^3}\bigg|_{x=x_n} > 0. \tag{4}$$

The total energy $U(x)$ of our system is the sum of the energy of the chemical bond $U_{\text{chem}}(x)$ and the energy of the resonator that can be taken as an ideal harmonic oscillator. When the system works at x_n, the total force $F(x)$ can be expanded near x_n as:

$$F(x) \approx -\eta(x - x_n)^2 - k_0(x - x_n). \tag{5}$$

Here k_0 the natural spring constant of the resonator and $\eta = (1/2)\partial^3 U_{\text{chem}}/\partial x^3$ from the chemical interaction. The physics is clear: we simply move the resonator to make it equilibrate at x_n. It is noted that there is another unstable equilibrium point $x_m = x_n - k_0/\eta$ with the total energy being local maximum, and the energy barrier between x_m and x_n is $\Delta E = (5/6)k_0^3/\eta^2$. Generally speaking, at finite temperature T, the system working at the local stable point x_n can escape over x_m[14]. To make the probability of such events small enough, it is required that $\Delta E \gg (1/2)k_BT$, which leads to the following condition on the resonator's natural spring constant

$$k_0 \gg \sqrt[3]{\frac{6k_BT\eta^2}{5}}. \tag{6}$$

The system's instability is expressed by the rate of escape κ_{es}, which is estimated as[13, 14]:

$$\kappa_{es} = \frac{\omega_c\omega_0}{\kappa}e^{-\frac{\Delta E}{k_BT}}. \tag{7}$$

Here ω_0 is the natural frequency of the resonator, $\omega_c = \sqrt{\frac{\partial F(x)}{m\partial x}|_{x=x_m}}$ is the effective frequency at x_m, and $\kappa = \omega_0/Q$ is the decay rate, with Q being the quality factor of the resonator.

Modeling the nonlinear dynamics

The system under consideration is a doubly clamped beam with its central point coupled to a atom contact whose size are much smaller than the beam, the displacement of the beam at the

atom contact are denoted as x. By considering only the fundamental mode of the beam, we employ the following equation of motion to described the dynamics:

$$m\frac{\mathrm{d}^2x}{\mathrm{d}t^2}+\gamma\frac{\mathrm{d}x}{\mathrm{d}t}+m\omega_0^2x+\beta x^2+\alpha x^3=\xi F(t) \tag{8}$$

with ω_0 the resonance frequency, m the effective mass,γ the linear dissipation, which is related to resonator's quality factor Q as $\gamma = m\omega_0/Q$, β the quadratic nonlinear response, α the cubic nonlinear response (Duffing constant) of the system, and $F(t)$ the external uniformly applied force on the entire beam. An effective spring constant can be defined as $k = m\omega_0^2$. For the specific vibrational mode [15], $m = \int_{-l/2}^{l/2} \rho\phi(y)^2 dy$, with ρ the density and $\phi(y)$ the corresponding shape factor, with normalization condition $\phi(0) = 1$ being used, and l is the length of the beam. The shape factor $\xi = \int_{-l/2}^{l/2} \phi(y)dy$ is obtained using finite element simulation based on the commercial software COMSOL, from which the normalized shape $\phi(y)$ is calculated. For the first-order vibration mode of the beam and the atom contact weakly coupled (non-contact regime), $\xi \approx 0.5$ close to the value for the free beam case. While for the atom contact strongly coupled via chemical bond (contact regime), this value can be estimated by considering the deforming of the beam due to a point linear response boundary condition. By using the parameters from the specific experimental device, a typical value in our system is $\xi \approx 0.7$.

For the study of bi-states dynamics, the higher orders of nonlinearity are neglected. The value of the Duffing nonlinear response α is of interesting and can be divided as intrinsic one α_0 that is from the beam stretching and a chemical bonding force induced one denoted as α_{chem} that can be tuned.

For standard Duffing resonator with quadratic nonlinear response β zero, the system dynamics is well studied, for vibration value higher than a critical value x_c, bi-states emerged. In such bi-states regime, the frequency response under coherent drive is well studied and can be described as:

$$x(\omega)=\frac{\frac{\xi F}{2m\omega_0^2}}{\frac{\omega-\omega_0}{\omega_0}-\frac{3}{8}\frac{\alpha}{m\omega_0^2}\left|x(\omega)\right|^2+i\left(\frac{1}{2Q}\right)} \tag{9}$$

The critical force F_c that drives the system system into bi-states regime is directly given by:

$$F_c=\left(\frac{4}{3}\right)^{\frac{5}{4}}\frac{1}{\xi}\omega_0^3Q^{-\frac{3}{2}}\sqrt{\frac{m^3}{\alpha}} \tag{10}$$

and the corresponding power dissipation into the environment is given by

$$P_c=\frac{3}{\xi}(1+\frac{\sqrt{3}}{2Q})\frac{16}{9}\frac{m^2\omega_0^5}{\alpha Q^2} \tag{11}$$

The quadratic nonlinear response β in our case is normally nonzero, and this makes our system a non-ideal Duffing nonlinear system. Simulation results in Supplementary Figure **3** show

that, for the values of β that are close to the non-contact regime where the bi-states dynamics are studied, the influence changes neither the frequency response nor the bi-states dynamics significantly greatly from the ideal Duffing one. At higher values, such difference may become important, which however, beyond our current experimental regime. Therefore we simplify the equation to a standard Duffing nonlinear one as:

$$m\frac{\mathrm{d}^2x}{\mathrm{d}t^2}+\gamma\frac{\mathrm{d}x}{\mathrm{d}t}+m\omega_0^2x+\alpha x^3=\xi F(t), \tag{12}$$

Definition and Modeling of the nonlinear response

To define the nonlinear response, we express the total potential energy of the system as:

$$U(x)=U_{\text{beam}}(x)+U_{\text{chem}}(x)-\xi Fx, \tag{13}$$

where x and ξ are the displacement of the central point of the beam and the shape factor as described above, $U_{\text{beam}}(x)$ is the beam's intrinsic elastic potential energy and is taken approximately as harmonic. $U_{\text{chem}}(x)$ is the interaction energy of the atom contact. To expand $U(x)$ at the position of equilibrium x_{eq}, we denote the vibrational part as $x_{\text{ac}}=x-x_{\text{eq}}$. We have the form:

$$U(x)=U_0(x_{\text{eq}})+\frac{1}{2}k(x_{\text{eq}})x_{\text{ac}}^2+\frac{1}{3}\beta(x_{\text{eq}})x_{\text{ac}}^3+\frac{1}{4}\alpha(x_{\text{eq}})x_{\text{ac}}^4+\text{ high-order terms.} \tag{14}$$

It is noted that the first order term in expanding (Supplementary Equation 14) is zero on condition of force being balanced, i.e.,

$$\frac{\partial U(x_{\text{eq}})}{\partial x_{\text{eq}}}=0, \tag{15}$$

which determines x_{eq} as a function of applied force F via $\xi F=\partial U(x_{\text{eq}})/\partial x_{\text{eq}}$. The higher orders of the expansion are dropped. The β and α are defined as quadratic nonlinear constant and cubic (Duffing) nonlinear response from above expansion. For the limit of free beam without chemical force interaction, the nonlinear responses thus defined only differ by a constant (not far from 1) from what are usually adopted [16].

In the presence of change in position of equilibrium δx_{eq}, the effective spring contact k shifts by a value Δk, which can be obtained from measured frequency shift δf as

$$\Delta k=2k\frac{\delta f}{f}, \tag{16}$$

with f being the resonance resonance frequency corresponding to beam in x_{eq}.

The quadratic nonlinear constant $\beta(x_{\text{eq}})=(1/2)\partial^3U/\partial x_{\text{eq}}^3$ can be further expressed as a function of the applied force F by utilizing the relations $k(x_{\text{eq}})=\partial^2U/\partial x_{\text{eq}}^2$ and $\xi F(x_{\text{eq}})=\partial U/\partial x_{\text{eq}}$, i.e.,

$$\beta=\frac{1}{2}\frac{\partial k(x_{\text{eq}})}{\partial x_{\text{eq}}}=\frac{1}{2}\frac{\partial k(F)}{\partial F}\frac{\partial F}{\partial x_{\text{eq}}}=\frac{1}{2\xi}\frac{\partial k(F)}{\partial F}k. \tag{17}$$

Hence, β can be obtained from the dependence of measured frequency of the resonator on the applied control force when the resonator is working at linear response regime. Similarly, the Duffing constant α can be derived as

$$\alpha = \frac{1}{6\xi^2}\frac{\partial^2 k}{\partial F^2}k^2 + \frac{1}{6\xi^2}k\left(\frac{\partial k}{\partial F}\right)^2. \tag{18}$$

Comparison of different methods of generating mechanical nonlinearity

Supplementary Table **1** tabulates various methods to generate nonlinear response in mechanical system reported in the literature. For comparison, we theoretically estimated the electrostatic interaction based double clamped beam strcuture[2] similar to our system by using a geometry dimension of the beam that is exactly the same as ours. Finite elements simulations based on commercial software COMSOL combined with quasi-analytic calculations are employed. The bias voltage V_{bias} is so chosen that the electrostatic interaction is maximum while the beam remains suspended without being attracted to the contact electrode. The results are shown in Supplementary Figure **4**.

Supplementary Note 2 | Experimental description

Fabrication of the device

The device in this experiment is composed of a suspended mechanical beam coupled to an gold-atom contact. It is fabricated using the standard nanolithography based on commercial Silicon-On-Insulator (1.5 μm Si /1 μm SiO_2). Large electrode pads are defined by photolithography and metal evaporation. To minimize the total resistance, we first put on a relative thin layer of gold electrodes (40 nm), and then do a second evaporation of a thick layer of gold electrodes (120 nm) using a slightly different mask. The beam area and the nanowire nearby is patterned using e-beam writer. Multi-layer metal is evaporated onto the exposed area as a conducting layer and silicon-etching mask. The unwanted metal is lift-off in acetone. Then the exposed silicon without metal cover is etched by inductively coupled plasma. Finally, the mechanical structure is suspended using a Buffered HF wet etch. The conducting metal layer above the silicon is consisted of 10 nm Cr, 80 nm Au and then 10 nm Cr. The nanobridge connecting the beam and the electrode is about 200 nm long and 80 nm wide, and is narrowed by focus-ion beam (FIB) to less than 50 nm. Once such a device of doubly clamped beam with a suspended nano-bridge has been successfully fabricated, it is placed in the ultrahigh vacuum environment of a 4 K cryogenic system, and then the bridge is electromigrated to form an atomic point contact. The electromigration procedure is similar as described in reference[17].

Parameters of the circuit and device

The measurement circuit is shown in Supplementary Figure 5A, and the parameter are list below:

Electric circuit:

$R_m = 191\ \Omega$
$C_m = 1.6\ \mu\text{F}$
$L_m = 6.4\ \text{nH}$
$R_b = 87\ \Omega$
$R_0 = 3.9\ \text{k}\Omega$
$R_1 = 20\ \Omega$
$R_2 = 50\ \text{k}\Omega$
$C_0 = 27\ \text{nF}$
$C_1 = 15\ \text{pF}$
$C_2 = 2.2\ \text{pF}$
$C_B = 10\ \text{nF}$
$L_B = 465\ \mu\text{H}$

R.f. source phase noise:

$S_\phi \leq -94\ \text{dBc@100 Hz}$
$Z_{out} = 50\ \Omega$

Voltage preamplifier:

$S^{\text{V}}_{\text{im}} = 4.0\ \text{nV}/\sqrt{\text{Hz}}$
$S^{\text{I}}_{\text{ba}} = 6.5\ \text{pA}/\sqrt{\text{Hz}}$
$Z_{in} = 1\ \text{M}\Omega || 20\ \text{pf}$

Mechanical resonator (used for measuring Figure 4 of main text):

$l = 50\ \mu\text{m}$
$t = 1.5\ \mu\text{m}$
$w = 0.51\ \mu\text{m}$
$k_0 = 10\ \text{N/m}$
$\omega_0/2\pi = 1.58\ \text{MHz}$
$Q = 3100$

The measurement voltage imprecision S^{V}_{im} is measured by shorting the input of voltage preamplifier and using 1 Hz bandwidth, and the current leakage S^{I}_{ba} is measured by connecting a 680 pf capacitor to the input of the voltage preamplifier using 1 Hz bandwidth. Then the current leakage can be calculated by using our equivalent noise model of the amplifier, and the effective noise temperature of the input of voltage preamplifier is about 470 K.

Calibration and displacement stability of the system

We take the mechanical resonator as equivalent to electric elements R_m, C_m and L_m by following the standard procedure[18] as shown in Supplementary Figure 5B. To extract the signal of

displacement from the measured voltage in input of the preamplifier, in frequency domain with frequency ω, we denote the input voltage to be V_{in} which is complex and in our experiments, we used the real part denoted as $V_{\text{in}}^{\text{Re}}$. By solving the measurement circuit, we can get the effective amplitude of the voltage generated from the vibrating resonator in magnetic field V_{eff}. In our case, $V_{\text{eff}} \approx \epsilon V_{\text{in}}^{\text{Re}}$ with coefficient $\epsilon = 0.9$, and then the mechanical vibration amplitude is simply related to V_{eff} as[18]:

$$x = \frac{V_{\text{eff}}}{\xi B l \omega}. \tag{19}$$

In our system, the transduction between $V_{\text{in}}^{\text{Re}}$ and x is about $3\ \mu\text{V}/\text{nm}$. In our experiment, we only drive the resonator into the nonlinear regime not very far from the critical point, so that it leads only to a small bias of calculated displacement, whose absolute value is not important in our experiments. Since the equivalent impedance of mechanical part is at least one order of magnitudes smaller than the total impedance in the circuit, such a linear approximation does not change the electronics noise estimation which is of importance in our experiments.

The force exerted on the beam equals to BlI, where I is the current pass through the beam. By solving the circuit, we can relate the driving voltage amplitude V_{drive} to the force with the transduction coefficient about 1.8 fN/μV.

We carried out finite element simulation to estimate the natural spring constant k_0. The calculated frequency agrees well with the measured natural frequency, indicating that the internal strain is small. The sample stage's temperature is 5.5 K. The Joule heating due to d.c. current that used to generate the force to tune the beam's position is obtained by using finite element simulation. The temperature increase at the surface of the sample chip close to the resonator is about 0.5 K. So the environment temperature of the resonator of 6 K is taken to be the estimation of thermal force noise and thermal motion.

Stability of the atom contact

The change in the equilibrium position of the atom contact is important in our experiments. We obtain this by recording the frequency drift and using the follow relation:

$$\delta x_{\text{eq}} \approx \frac{\delta\omega}{k(\partial\omega/\partial F)}. \tag{20}$$

Here, the coefficient $\partial\omega/\partial F$ is experimentally measured, with Supplementary Figure **6** showing the results of 5000 data for a typical device. The standard deviation is obtained to be 1.2 pm. In a typical experiments process (less than 1 minutes), smaller drifts are expected.

When working in non-contact regime, the beam could be attracted to contact the electrode as described above. If this occurs, we can usually use a large lorentz force to pull the beam out of touch again, but such a process can lead to structural change in the atom contact. Supplementary Figure **7** shows a typical dependence of the frequency and α (of device E) on the control force in non-contact regime before and after such jump-to-contact.

Intrinsic nonlinearity of the resonator

The intrinsic Duffing nonlinearity coefficient α_0 is measured from the frequency response under various driving strengths. When doing this, we have pulled the beam far away from the atom contact (more than 30 nm) so that the atom contact interaction is negligible. Based on the model above (Supplementary Note 1), we swept the frequency from low to high, and recorded the max vibration amplitude x_{max} as well as the corresponding frequency ω_{max}. The measured data for a typical device are shown in Supplementary Figure **8**A. There is a relation:[16]

$$\omega_{\text{max}} = \omega_0 + \frac{3}{8}\frac{\alpha_0}{m\omega_0}(x_{\text{max}})^2. \tag{21}$$

The nonlinearity can be obtained by fitting measured data to the above relation as shown in Supplementary Figure **8**B.

Magnetic field induced damping

The magnetic based drive and measurement scheme could leads to dissipation[18] whose strength scales as Supplementary Figure **9**. For a typical device such effects are observed by measuring the magnetic field dependent quality factor. In our system, the quality factor decreases by about a factor of 10 in value as magnetic goes to zero due to magnetic damping.

Influence of electrostatic force

In the non-contact regime, long-range electrostatic force may also contribute to the total mechanical property and is expected to be proportional to the bias between the contact. The mechanical resonance frequency shift as a function of bias applied on the contact for a typical device is plotted in Supplementary Figure **10**A. The frequency shift and nonlinear response of the device as functions of the applied external force for the bias 0 mV and -200 mV almost coincide. For our measurements presented in this work, we adopted the bias -200 mV to minimized the frequency shift due to electrostatic effect.

Supplementary Note 3 | Electronic noise

Noise from measurement circuit

The measurement circuit brings in electronic noise which leads to current noise through the beam, and so it generates mechanical noise via Lorentz force. We denote this current noise by power density $S^{\text{I}}_{\text{mea}}$, and then the corresponding force noise can be estimated as:

$$S^{\text{F}}_{\text{mea}} = S^{\text{I}}_{\text{mea}} B^2 l^2. \tag{22}$$

There are mainly three sources that contribute to $S_{\mathrm{mea}}^{\mathrm{I}}$. The first is the Johnson-Nyquist noise of resistance in circuit, which is directly equivalent to a voltage source with value $S_{\mathrm{R}}^{\mathrm{V}} = 4Rk_BT$, with T the room temperature and R the electric resistance. The second is the current leakage from the input of the preamplifier $S_{\mathrm{ba}}^{\mathrm{I}}$. We denote this as back-action noise and model it by a current source following reference[19]. In doing this, we assume that there is no correlation between the voltage imprecision $S_{\mathrm{im}}^{\mathrm{V}}$ and the back-action $S_{\mathrm{ba}}^{\mathrm{I}}$. The third noise is from the d.c. bias that is generated in our experiment by an isolated voltage source, with the signal being filtered, so that its influence can be neglected.

We solve the equivalent circuit in Supplementary Figure 5B and obtain the expression of the total current noise as:

$$S_{\mathrm{mea}}^{\mathrm{I}}(\omega) = |A_1|^2 S_{\mathrm{ba}}^{\mathrm{I}}(\omega) + |A_2|^2 S_0^{\mathrm{V}}(\omega) + |A_3|^2 S_1^{\mathrm{V}}(\omega) + |A_4|^2 S_{\mathrm{b}}^{\mathrm{V}}(\omega) \tag{23}$$

with coefficients A_1, A_2,A_3 and A_4 being decided by the circuit. As a result, the current noise $\sqrt{S_{\mathrm{mea}}^{\mathrm{I}}}$ in our experiment is about $170\ \mathrm{fA} \cdot \mathrm{Hz}^{-1/2}$, with the corresponding force noise $\sqrt{S_{\mathrm{mea}}^{\mathrm{F}}} = 51\ \mathrm{aN} \cdot \mathrm{Hz}^{-1/2}$.

Phase noise in driving signal

The phase noise works effectively as mechanical noise which could drive nonlinear switching dynamics if it is strong enough[20]. To estimate this effect, we convert the phase noise into force noise by considering the output signal of r.f. source as[21]:

$$V_{\mathrm{out}} = V_0 \cos[\omega_0 t + \phi(t)]. \tag{24}$$

Here V_0 is the output amplitude and $\phi(t)$ is phase fluctuation with its average being zero, i.e.,

$$R_{\psi,\psi}(\tau) = \overline{e^{j\omega_0\tau}[1 + \phi(t)\phi(t+\tau)]}. \tag{25}$$

This leads to a power density $S_\psi(\omega)$ via relation

$$S_\psi(\omega) = \int_{-\infty}^{+\infty} R_{\psi,\psi}(\tau) e^{-i\omega\tau} \mathrm{d}\tau \tag{26}$$

of the following form:

$$S_\psi(\omega) = 2\pi\delta(\omega - \omega_0) + S_\phi(\omega - \omega_0). \tag{27}$$

The equivalent output power density of voltage takes the form:

$$S_{\mathrm{V}}(\omega) = V_0^2 \left[2\pi\delta(\omega - \omega_0) + S_\phi(\omega - \omega_0).\right] \tag{28}$$

The first term in the right hand side corresponds to the idea r.f. output and the second term is an effective voltage noise. It commonly has a Lorentz shape[22]. Also, there is an output noise from the r.f. source with strength $25\,\mathrm{nV}\cdot\mathrm{Hz}^{-1/2}$, all these noises are included by a power density, denoted as S_0^{V} in the circuit (Supplementary Figure 5). With $V_0 = 2\mathrm{mV}$ in our experiment, the voltage noise applied on the beam in bandwidth of interesting is about $39\ \mathrm{nV}\cdot\mathrm{Hz}^{-1/2}$, so that the corresponding force noise is about $46\ \ \mathrm{aN}\cdot\mathrm{Hz}^{-1/2}$.

Noise from the atom contact

Two sources of noise contribute to the force noise of the resonator, with the first being the electrostatic force fluctuation from the applied voltage, and the second being from the tunneling current in the atom contact. To suppress these noises, we applied in our experiments a $-200\,\mathrm{mV}$ voltage onto the stiff electrode to minimize the electric force, with uncertainty less than 10 mV. The power density of the voltage fluctuation of the noise isolated voltage source used is much less than $1\ \ \mu\mathrm{V}\cdot\mathrm{Hz}^{-1/2}$ in the frequency range of interest, so that the corresponding electrostatic force has a power density much less than $20\ \ \mathrm{aN}\cdot\mathrm{Hz}^{-1/2}$. For the second potential contribution, the tunneling induced force on the mechanical resonator has been well understand[23]. It is generated from the momentum transfer of tunneling electrons and can be expressed as:

$$S_{\mathrm{tun}}^{\mathrm{F}} = \frac{2p_f^2 V_{\mathrm{bias}}}{eR_{\mathrm{tun}}}. \tag{29}$$

Here V_{bias} is taken to be $10\ \mathrm{mV}$ and p_f is the electron fermi energy of gold. In our experiments, the tunneling resistance R_{tun} is measured using a picoammeter, and the measured value is larger than $1\ \mathrm{M}\Omega$ in the non-contact regime where the measurement of thermal induced bi-states dyanmics (Supplementary Figure 4 in main text) is performed. In the experiment, however, we replaced the picometer by a low noise isolated voltage source. The force noise from tunneling is less than $1\ \mathrm{aN}\cdot\mathrm{Hz}^{-1/2}$ and so is also negligible. Noise strengths are listed in Supplementary Table 2.

Supplementary References

[1] Suh, J., LaHaye, M. D., Echternach, P. M., Schwab, K. C. & Roukes, M. L. Parametric amplification and back-action noise squeezing by a qubit-coupled nanoresonator. *Nano Letters* **10,** 3990 (2010).

[2] Kozinsky, I., Postma, H. C., Bargatin, I., & Roukes, M. L. Tuning nonlinearity, dynamic range, and frequency of nanomechanical resonators. *Appl. Phys. Lett.* **88,** 253101 (2006).

[3] Lee, S. I., Howell, S. W., Raman, A., & Reifenberger, R. Nonlinear dynamics of micro-cantilevers in tapping mode atomic force microscopy: A comparison between theory and experiment. *Phys. Rev. B.* **66,** 115409 (2002).

[4] Chan, H. B., Aksyuk, V. A., Kleiman, R. N., Bishop, D. J., & Capasso, F. Nonlinear micromechanical Casimir oscillator. *Phys. Rev. Lett.* **87,** 211801 (2001).

[5] Sankey, J. C., Yang, C., Zwickl, B. M., Jayich, A. M., & Harris, J. G. Strong and tunable nonlinear optomechanical coupling in a low-loss system. *Nature Phys.* **6,** 707 (2010).

[6] Karabalin, R. B. *et al.* Signal amplification by sensitive control of bifurcation topology. *Phys. Rev. Lett.* **106,** 094102 (2011)

[7] Eichler, A., Moser, J., Chaste, J., Zdrojek, M., Wilson-Rae, I., & Bachtold, A. Nonlinear damping in mechanical resonators made from carbon nanotubes and graphene. *Nature Nanotech.* **6,** 339 (2011).

[8] Israelachvili, J. N. *Intermolecular and Surface Forces with Applications to Colloidal and Biological Systems* (Academic Press, New York, 1985)

[9] Kresse, G. & Hafner, J. Norm-conserving and ultrasoft pseudopotentials for first-row and transition-elements. *J. Phys.: Condens. Matter* **6,** 8245 (1994).

[10] Perdew, J. P., Burke, K. & Ernzerhof, M. Generalized gradient approximation made simple. *Phys. Rev. Lett.* **77,** 3865 (1996).

[11] Blöchl, P. E. Projector augmented-wave method. *Phys. Rev. B* **50,** 17953 (1994).

[12] Kresse, G. & Joubert, D. From ultrasoft pseudopotentials to the projector augmented-wave method. *Phys. Rev. B* **59,** 1758 (1999).

[13] Kramers, H. A. Brownian motion in a field of force and the diffusion model of chemical reactions. *Physica (Utrecht)* **7,** 284 (1940).

[14] Chandrasekhar, S. Stochastic problems in physics and astronomy. *Rev. Mod. Phys.* **15,** 1 (1943).

[15] Villanueva, L. G. Karabalin, R. B. Matheny, M. H. Chi, D. Sader, J. E. & Roukes, M. L. Nonlinearity in nanomechanical cantilevers. *Phys. Rev. B* **87,** 024304 (2013).

[16] Lifshitz, R. & Cross, M. C. *Review of Nonlinear Dynamics and Complexity* (Wiley-VCH, 2009).

[17] Park, H., Lim, A. K. L., Alivisatos, A. P., Park, J. & McEuen, P. L. Fabrication of metallic electrodes with nanometer separation by electromigration. *Appl. Phys. Lett.* **75,** 301 (1999).

[18] Cleland, A. & Roukes, M. External control of dissipation in a nanometer-scale radiofrequency mechanical resonator. *Sensors and Actuators A* **72,** 256-261 (1999).

[19] Devoret, M. H. & Schoelkopf, R. J. Amplifying quantum signals with the single-electron transistor. *Nature* **406,** 1039-1046 (2000).

[20] Aldridge, J. S. & Cleland, A. N. Noise-enabled precision measurements of a Duffing nanomechanical resonator. *Phys. Rev. Lett.* **94,** 156403 (2005).

[21] Cleland, A. & Roukes, M. Noise processes in nanomechanical resonators. *J. Appl. Phys.* **92,** 2758-2769 (2002).

[22] Milotti, E. Amplitude to phase noise conversion in electronic circuits. *Phys. Rev. E* **57,** 67 (1998).

[23] Bocko, M. F., Stephenson, K. A. & Koch, R. H. Vacuum tunneling probe: a nonreciprocal, reduced-back-action transducer. *Phys. Rev. Lett.* **61,** 726 (1988).

[24] Giessibl, F. J. Advances in atomic force microscopy. *Rev. Mod. Phys.* **75,** 949-983 (2003).
